# Supplementary material for: Study on the China’s real interest rate after including housing price factor into CPI
Source: PLoS One. 2023 Aug 11;18(8):e0290079. doi: 10.1371/journal.pone.0290079 (PMC10420356; doi:10.1371/journal.pone.0290079)
Supplement: Attachment — Submitted filename: Response to editor 0614.docx [file pone.0290079.s002.docx]

Dear Maidelyn R. Peregrin,

Thank you for your recommends on our manuscript. We have revised the manuscript accordingly as follow:

1. The titles of Figure 1, 2, 3 and 4 were added in proper positions;
2. The data source were revised as follow in footnote 1 with the data link:

Data source: “China Real Estate Statistical Yearbook” “China Statistical Yearbook” “China Financial Statistical Yearbook”, and the all of those Yearbook can be found in “China Statistical Yearbooks Database” with the link: <https://data.cnki.net/.>

Kindly regards

Ding Shiting on behalf of all authors

14/06/2023
